# Larval morphology of *Panorpodes kuandianensis* (Insecta, Mecoptera, Panorpodidae) and its evolutionary implications

**DOI:** 10.3897/zookeys.398.6675

**Published:** 2014-04-04

**Authors:** Lu Jiang, Chao Yue, Baozhen Hua

**Affiliations:** 1 State Key Laboratory of Crop Stress Biology for Arid Areas, Key Laboratory of Plant Protection Resources and Pest Management of the Education Ministry, Northwest A&F University, Yangling, Shaanxi 712100, China; 2 Department of Life Science and Technology,; 3 Nanyang Normal University, Nanyang, Henan 473061, China

**Keywords:** Chaetotaxy, evolution, homology, larva, mouthparts, proleg

## Abstract

Larval characters play a significant role in evolutionary and systematic studies of holometabolous insects. However, Panorpodidae, a derived family of Mecoptera, are largely unknown in their immature stages to date. Here, the first instar larva of the short-faced scorpionfly *Panorpodes kuandianensis* Zhong, Zhang & Hua, 2011 is described and illustrated using light and scanning electron microscopy. The larva of *Panorpodes* is remarkable for the absence of compound eyes on the head and the presence of seven small unpaired proleg-like processes along the midventral line on abdominal segments II–VIII. The homology of these unpaired appendage-like processes, their ecological adaptation, and the evolutionary implications of some larval characters of Panorpodidae are discussed.

## Introduction

The larva is an important developmental stage of insects in Endopterygota (= Holometabola) (Grimaldi and Engel 2005; Van Emden 1957; Zacharuk and Shields 1991), the most successful lineage in terrestrial animals (Kristensen 1999). The larvae are dramatically divergent in external morphology and food habits, and frequently occupy different ecological niches and habitats from their adults (Yang 2001). However, the evolutionary origin of insect larvae remains controversial (Hall and Wake 1999).

The Mecoptera are one of the primitive lineages in the Endopterygota, with the fossil record dated from lower Permian to Mesozoic (Byers and Thornhill 1983; Grimaldi and Engel 2005). The larvae of Panorpidae and Bittacidae are eruciform, bearing eight pairs of abdominal prolegs in addition to three pairs of thoracic legs. The prolegs are considered nonhomologous with the thoracic legs in Panorpidae, and different from other eruciform larvae in Lepidoptera and Hymenoptera (Du et al. 2009; Yue and Hua 2010). However, the larvae of Panorpodidae, the sister group of Panorpidae (Willmann 1987), have not been thoroughly investigated.

Panorpodidae consist of 13 described species distributed disjunctly in the Northern Hemisphere and are assigned to two genera (Zhong et al. 2011). *Panorpodes* MacLachlan, 1875 occurs in China, Korea, Japan, and western North America (Byers 2005; MacLachlan 1875; Tan and Hua 2008b; Zhong et al. 2011). *Brachypanorpa* Carpenter, 1931 is distributed exclusively in eastern North America (Byers 1997; Carpenter 1931b, 1953). The phylogenetic position of Panorpodidae in Mecoptera remains controversial between molecular and morphological evidence (Pollmann et al. 2008). The molecular evidence suggests that the sister group of Panorpodidae is Bittacidae (Whiting 2002), while morphological studies demonstrate a sister relationship between Panorpodidae and Panorpidae (Friedrich et al. 2013; Willmann 1987, 1989). Based on biological and morphological characters, Penny (1977) even concluded a close relationship between Panorpodidae and Boreidae. Detailed studies on larval morphology may provide additional or even crucial evidence for the phylogenetic analysis of Mecoptera (Beutel et al. 2009).

The knowledge of Panorpodidae larvae is far from satisfactory largely owing to the restricted species distribution and the mysterious larval diets (Byers 1997; Byers and Thornhill 1983; Carpenter 1931a, 1953; Zhong et al. 2011). The larvae of the North American *Brachypanorpa* are eyeless and lack prolegs on abdominal segments, and are regarded as scarabaeiform (Byers 1997), although a small cylindrical structure is present mid-ventrally on each abdominal segments III–VI of the larva. Suzuki (1985, 1990) successfully obtained the first instar larva of *Panorpodes paradoxa* in his embryological study, but provided no detailed description, such that the knowledge of larval *Panorpodes* still remains largely unknown.

In this study, we investigated the larvae of the short-faced scorpionfly *Panorpodes kuandianensis* Zhong, Zhang & Hua, 2011 through rearing, and illustrated the first instar larvae using light and scanning electron microscopy, in an attempt to acquire more evidence for the larval evolutionary study of Mecoptera.

## Materials and methods

Adults of *Panorpodes kuandianensis* were captured from Huaboshan (41°06'N, 125°02'E, elev. 650–1100 m), Kuandian County, Liaoning Province of northeastern China from late June to July in 2011 and 2012. The adults were reared in pairs in plastic jars covered with a piece of gauze. Humid soil (5 cm in depth) covered with moss was placed at the bottom of the jar for adults resting and oviposition. Fresh leaves, flowers and honey drops were daily provided as potential food items.

First instar larvae were fixed in Carnoy’s fixative solution (95% ethanol: glacial acetic acid = 3:1, v/v) for 12 h before being preserved in 75% ethanol. After dehydration in a graded ethanol series (75%, 85%, 95%, 100%), the samples were transferred to isoamyl acetate twice for 30 min, critical-point dried with liquid carbon dioxide, sputter-coated with gold, and examined in a Hitachi S-3400N scanning electron microscope (Hitachi, Tokyo, Japan) at 15 kV.

To illustrate chaetotaxy, SEM photographs were taken for each segment of the first instar larva on dorsal, lateral and ventral surfaces, respectively. Draft drawings were improved with Adobe Illustrator CS4.

## Results

### General morphology of the larva

**Figure 1.**
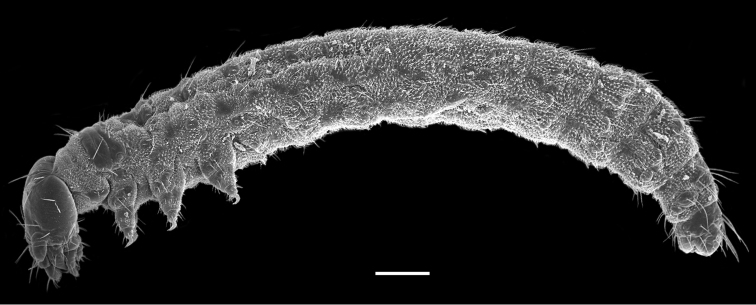
First instar larva of *Panorpodes kuandianensis*. Scale bar = 200 µm.

The first instar larva is white and 2.9 ± 0.31 mm in length (*n* = 10) (Fig. 1). The head is hypognathous and eyeless, with mandibulate mouthparts directed downward and a pair of three-segmented antennae lateroventrally. The trunk is cylindrical and furnished with numerous cuticular spinules and setiform setae. The thorax possesses three pairs of legs. The abdomen has eleven segments and possesses seven unpaired appendage-like processes mid-ventrally on each of abdominal segments II–VIII. The respiratory system is peripneustic, with one pair of spiracles on the prothorax and eight pairs of spiracles on the first eight abdominal segments. The telson bears a protrusile sucker.

### Head capsule

**Figure 2.**
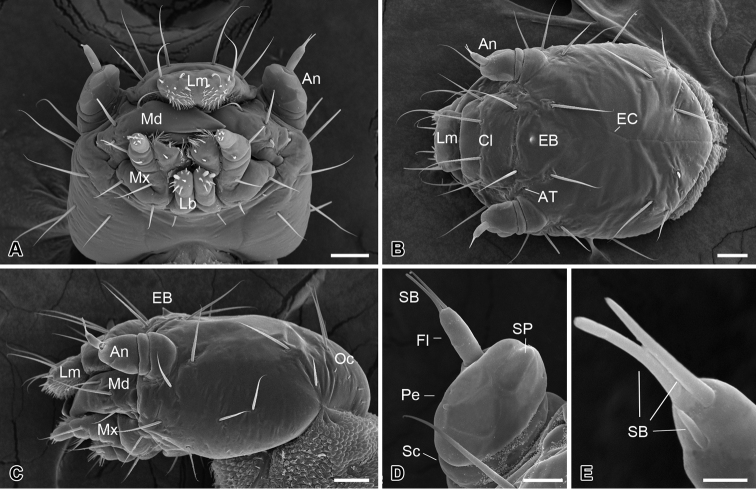
Larval head of *Panorpodes kuandianensis*. **A** Ventral view **B** Dorsal view **C** Lateral view **D** Antenna (ventral view) **E** Sensilla on flagellum (dorsal view). Abbreviations: **An** = antenna, **AT** = anterior tentorial pit, **Cl** = clypeus, **EB** = egg burster, **EC** = ecdysial cleavage, **Fl** = flagellum, **Lb** = labium, **Lm** = labrum, **Md** = mandible, **Mx** = maxilla, **Oc** = occiput, **Pe** = pedicel, **SB** = sensillum basiconicum, **Sc** = scape, **SP** = sensillum placodeum. Scale bars: (**A**)−(**C**) = 50 μm, (**D**) = 20 μm, (**E**) = 5 μm.

The head is slightly flattened, 450 ± 15 µm in length and 315 ± 17 µm in width (*n* = 10) (Fig. 2A–C), lacking compound eyes, ocelli, or stemmata (Fig. 2C). The frons is subtriangular and is confined by two ecdysial cleavage lines and a frontoclypeal sulcus (Fig. 2B), bearing centrally a sharp egg burster, which aids in hatching of the larva (Fig. 2B, C). A pair of anterior tentorial pits is situated at the lateral corners of the frons (Fig. 2B). Thirteen pairs of setiform setae are present on the head capsule symmetrically (Fig. 2A–C). Additionally, four pairs of minute setae occur on the occiput (Fig. 2C).

### Antennae

The antennae are three-segmented, each consisting of a basal scape, a pedicel, and a slender flagellum (Fig. 2D). The scape is very short and inserted into a prominent antennal socket. The pedicel is stout and slightly conical, five times longer than the scape, with ten sensilla placodea on the ventral surface. The distal flagellum is slender, inserted on the lateral apex of the pedicel, and bears apically one short and two long sensilla basiconica (Fig. 2E).

### Mouthparts

The mouthparts are of the mandibulate type (Fig. 2A), consisting of a labrum, a pair of mandibles, a pair of maxillae, and a labium.

The labrum is roughly trapezoid, articulated proximally with the anterior region of the clypeus (Fig. 2B). The labrum bears two pairs of apical setae, with the inner pair nearly half length of the outer pair (Fig. 2B).

**Figure 3.**
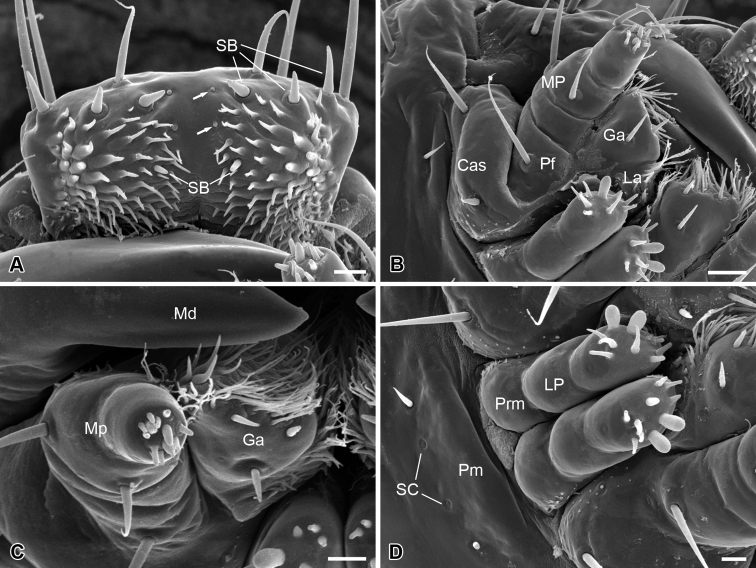
Larval mouthparts of *Panorpodes kuandianensis*. **A** Epipharyx, arrows show the inconspicuous sensilla basiconica **B** Maxilla (ventral view) **C** Maxilla (frontal view) **D** Labium. Abbreviations: **Cas** = cardo-stipes, **Ep** = epipharynx, **Ga** = galea, **La** = lacinia, **Lb** = labium, **LP** = labial palpus, **Md** = mandible, **MP** = maxillary palpus, **Pf** = palpifer, **Pm** = postmentum, **Prm** = prementum, **SB** = sensillum basiconicum, **SC** = sensillum campaniformium. Scale bars: (**A**), (**C**) and (**D**) = 10 μm, (**B**) = 20 μm.

The epipharynx is situated on the inner surface of the labrum (Fig. 3A), with three pairs of sensilla basiconica along the apical margin, a pair of short sensilla basiconica and two pairs of inconspicuous sensilla basiconica on the central part. The epipharynx is also furnished with sparse short microtrichia pointed inward at the lateral part, but lacks microtrichia along the middle axis.

The mandible is highly sclerotized, with the sharp incisor incurved apically; the mandibles cross each other apically. Each mandible possesses three sensilla chaetica on the outer surface (Fig. 2A–C).

The maxilla consists of a cardo-stipes, a galea, a lacinia, and a three-segmented palp (Fig. 3B). The original cardo and stipes are fused into a cardo-stipes, which bears two sensilla chaetica. The galea possesses three sensilla basiconica ventrally and numerous microtrichia distally (Fig. 3B, C). The lacinia is greatly reduced and bears a cluster of microtrichia distally. The palpifer carries a long sensillum chaeticum on the ventral surface. The maxillary palp is three-segmented and bears two short sensilla chaetica on the lateral surface of the second joint and 12 sensilla basiconica on the apical surface of the third joint (Fig. 3C).

The labium is highly vestigial, with the ligula absent (Fig. 3D). The postmentum is merged with the head capsule, bearing a pair of short sensilla chaetica and a pair of sensilla campaniformia. The prementum is mesally separated and bears distally a pair of two-segmented labial palps. The distal segment of the labial palp bears two large papillary and eight conical sensilla basiconica on the apex. These sensilla are slightly varied from specimen to specimen, even asymmetrical bilaterally between the left and the right palp.

### Thoracic legs

**Figure 4.**
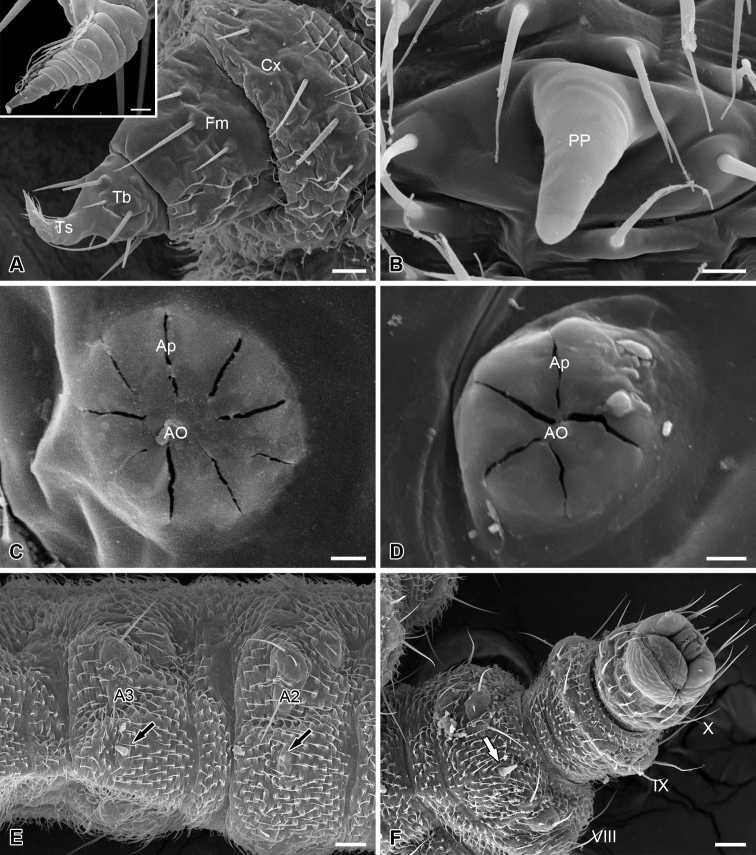
Thoracic leg, abdominal processes, spiracles and telson of the larva of *Panorpodes kuandianensis*. **A** Thoracic leg, inset shows magnification of the tarsus of thoracic leg **B** Proleg-like abdominal process **C** Prothoracic spiracle **D** Abdominal spiracle **E** Ventral view of abdominal segments II and III **F** Telson (ventral view). Abbreviations: **AO** = atrial orifice, **Ap** = aperture, **Cx** = coxa, **Fm** = femur, **PP** = proleg-like process, **Tb** = tibia, **Ts** = tarsus. Scale bars: (**A**) = 20 μm, (**B**) = 5 μm, (**C**) and (**D**) = 3 μm, (**E**) = 40 μm, (**F**) = 50 μm.

The thoracic legs are four-segmented, each consisting of a coxa, a femur, a tibia, and a tarsus (Fig. 4A). The coxa and femur are sclerotized on the anterior surface but membranous on the posterior surface. The femur and tibia bear several microsetae. The tarsus is slender and curved cephalad, with a hirsute anterior surface and a wrinkled posterior surface (Fig. 4A).

### Spiracles

Nine pairs of spiracles are located on the pleura of the larval trunk. The prothoracic spiracle is on the posterior corner of the prothoracic shield, with nine apertures surrounding the atrial orifice (Fig. 4C). Eight pairs of abdominal spiracles each are present on the pleura of the first eight abdominal segments, with 4–5 apertures (Fig. 4D).

### Abdomen

The abdomen consists of 11 segments and is furnished with numerous setiform setae and prominent cuticular spinules (Fig. 1). The larval abdomen bears seven inconspicuous unpaired mid-ventral processes on each A2–A8, with these smooth and unsegmented processes varying in length, greatly reduced on A2 (Fig. 4B, E, and F). The larval abdomen terminally bears a protrusile sucker, providing adhesive attachment during locomotion (Fig. 4F).

### Chaetotaxy of the larval trunk

**Figure 5.**
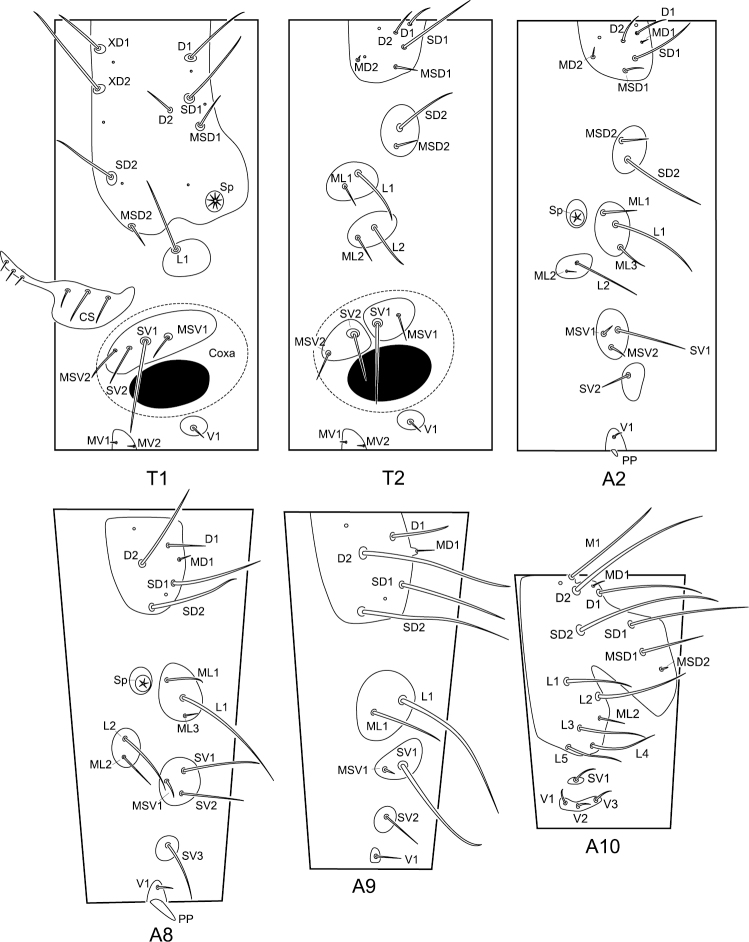
Chaetotaxy of the larval trunk of *Panorpodes kuandianensis*. Abbreviations: **CS** = cervical sclerite, **D** = dorsal seta, **L** = lateral seta, **M** = mid-dorsal seta, **MD** = minute dorsal seta, **MSD** = minute subdorsal seta, **MSV** = minute subventral seta, **MV** = minute ventral seta, **PP** = proleg-like process, **SD** = subdorsal seta, **Sp** = spiracle, **SV** = subventral seta, **V** = ventral seta, **XD** = prothoracic seta.

The meso- and metathorax are similar in chaetotaxy. Abdominal segments I–VII are similar in chaetotaxy (Fig. 5).

**Prothorax (T1).** The prothorax bears a prominent prothoracic shield, along the anterior margin of which are three long setae (XD1, XD2, and SD2) and one short seta (MSD2). Along the posterior edge of the shield are two long setae (D1 and SD1) and one short seta (MSD1). Below the shield is a long lateral seta (L1) alone on the lateral pinaculum. Two long setae (SV1 and SV2) and two short setae (MSV1 and MSV2) are on the subventral pinaculum. Mesal to the coxal cavity are one short ventral seta (V1) on a small pinaculum and a pair of minute setae (MV1 and MV2) on a midventral pinaculum.

**Meso- and metathorax (T2 and T3).** On the dorsal pinaculum are one long seta (SD1), three short setae (D1, D2, and MSD1), and one minute seta (MD2). On the subdorsal pinaculum are one long seta (SD2) and one short seta (MSD2). Two lateral pinacula each bear a long and a short seta (L1 and ML1, L2 and ML2). Two subventral pinacula each bear a long seta and a short seta (SV1 and MSV1, SV2 and MSV2). The ventral setae (V1, MV1, and MV2) exhibit a similar pattern to prothorax.

**Abdominal segments II–VII (A2–A7).** On the dorsal pinaculum are three long setae (D1, D2, and SD1) and three short setae (MD1, MD2, and MSD2). On the subdorsal pinaculum are one long and one short seta (SD2 and MSD2). On the lateral pinaculum posterior to the spiracle are one long (L1) and two short setae (ML1 and ML3). Another small lateral pinaculum below the spiracle bears a long (L2) and a short seta (ML2). One long (SV1) and two short setae (MV1 and MV2) are arranged on a subventral pinaculum. A short seta (SV2) is situated alone on another subventral pinaculum. The midventral pinaculum bears a short ventral seta (V1).

**Abdominal segment VIII (A8).** The dorsal pinaculum bears three long setae (D2, SD1, and SD2), one short seta (D1), and one minute seta (MD1). One long (L1) and two short setae (ML1 and ML3) are situated on the lateral pinaculum posterior to the spiracle. Another lateral pinaculum below the spiracle bears two setae (L2 and ML2). Two long setae (SV1 and SV2) and one minute seta (MSV1) are arranged on a subventral pinaculum, whereas a long seta (SV3) alone is located on another pinaculum. One short seta (V1) is situated on the midventral pinaculum lateral to the mid-ventral abdominal process (AP).

**Abdominal segment IX (A9).** On the dorsal pinaculum are three long setae (D2, SD1, and SD2) and one short seta (D1). On the lateral pinaculum are one long (L1) and one short seta (ML1). One long seta (SV1) is located on one subventral pinaculum. One short seta (SV2) is on another subventral pinaculum. A ventral seta (V1) is situated alone on the ventral pinaculum.

**Abdominal segment X (A10).** The epiproct bears one mid-dorsal seta (M1). Four long setae (D1, D2, SD1, and SD2) and one short seta (MSD1) are situated on the dorsal part of the tergum. Five long (L1–L5) and one short setae (ML2) are inserted on the pleuron. On the subventral pinaculum is one short seta (SV1). Three short setae (V1, V2, and V3) are arranged on the elongated narrow ventral pinaculum.

## Discussion

The larvae of Panorpodidae represented by *Panorpodes* are unique in Mecoptera for the absence of compound eyes on the head, presence of several unpaired midventral processes on A2−A8, and absence of erect subdorsal annulated processes on stout basal protuberances as in Panorpidae and Bittacidae (Chen and Hua 2011; Jiang and Hua 2013; Ma et al. 2014; Tan and Hua 2008a).

In Mecoptera the larvae are eruciform in Panorpidae, Choristidae, Apteropanorpidae, and Bittacidae (Byers 1991; Jiang and Hua 2013; Tan and Hua 2008a); campodeiform in Nannochoristidae (Pilgrim 1972); and scarabaeiform in Boreidae (Cooper 1974; Penny 1977; Russell 1982). The larvae of *Brachypanorpa* in Panorpodidae were also described as scarabaeiform (Byers 1997). Considering the presence of the unpaired midventral abdominal processes on the larvae of *Panorpodes* and *Brachypanorpa*, however, it is difficult to regard them as true scarabaeiform larvae.

In general, the larvae of Mecoptera are remarkable for the presence of a pair of compound eyes (Chen et al. 2012; Gilbert 1994; Melzer et al. 1994; Pilgrim 1972; Tan and Hua 2008a). The larval compound eye is composed of ten or more ommatidia in Nannochoristidae (Melzer et al. 1994; Pilgrim 1972), three “stemmata” in *Boreus* (Cooper 1974) and seven in *Caurinus* of Boreidae (Russell 1982), seven ommatidia (or "stemmata") in Bittacidae (Gilbert 1994; Tan and Hua 2008a), and approximately 20–40 ommatidia in Panorpidae (Boese 1973; Chen et al. 2012; Melzer 1994; Paulus 1979), representing a true plesiomorphy of Mecoptera in Endopterygota (Beutel et al. 2009). A dorsal ocellus is also present on the larval head of Bittacidae (Tan and Hua 2008a, 2009). The larvae of *Panorpodes*, however, are completely eyeless, congruent with the larvae of *Brachypanorpa* (Byers 1997). In fact, the visual organs (optic lobe) of *Panorpodes paradoxa* are present in the early embryonic stage, but are degenerate in later stages, and finally disappear by the end of embryonic revolution (Suzuki 1985), indicating this eyelessness is a secondary degeneration and represents an autapomorphy of Panorpodidae.

The larval prolegs of Panorpidae are formed by an inner pair of proleg primordia near the midventral line mesal to the true appendage primordia, and are not homologous with the thoracic legs (Yue and Hua 2010), confirming the hypothesis that prolegs are secondary adaptive structures (Hinton 1955). The presence of unpaired midventral processes in Panorpodidae larvae is difficult to explain by a recent hypothesis of coxal endite on the evolutionary origin of larval prolegs (Bitsch 2012). Because of the shared similarities (each process is delimited by the paired ventral setae, and these processes are varied in length with anterior one great reduced but posterior one longest) of Panorpodidae and Panorpidae, the unpaired midventral processes are likely homologous with and degenerated from the prolegs of the eruciform larvae in Panorpidae. The degeneration of larval prolegs as a rule was considered an evolutionary tendency in most Diptera, leaf-mining Lepidoptera, Coleoptera, and parasite Hymenoptera and Strepsiptera (Chapman 2013). In this case, the unpaired midventral processes may represent an advanced evolutionary stage of larval abdominal prolegs, and Panorpodidae may occupy a derived position in the phylogeny of Mecoptera.

The larvae of Panorpodidae lack dorsal protuberances on the first ten abdominal segments, distinctly divergent from those of Bittacidae and Panorpidae. In Bittacidae, the furcated protuberances borne on the dorsal surface of the larval trunk may assist adhering to soil particles as a camouflage (Tan and Hua 2008a). In Panorpidae, annulated protuberances are present on the larval trunk and are considered to keep the larval trunk from being injured in a subterresial life style (Ma et al. 2014). In Panorpodidae, the larvae of *Panorpodes kuandianensis* stay sedentary subterraneally with limited range of locomotion (L Jiang, unpublished data). We speculate that the absence of dorsal protuberances on the abdomen likely resulted from its inactive living habit in the soil, as in the soil-dwelling larvae in Scarabaeidae (Eilers et al. 2012).

The peculiar morphological characters of panorpodid larvae are likely related to their cryptic lifestyle. In the underground habitat, the larvae of Panorpodidae may reasonably use olfaction or gustation rather than vision as their sense organs. This situation is similar to the eyeless soil-dwelling larvae in Scarabaeidae (Eilers et al. 2012). Likewise, the larvae of Panorpodidae no longer need paired abdominal prolegs to support the abdomen and serve the locomotory function as in the larvae of Panorpidae (Yue and Hua 2010), thus their prolegs are reduced to vestigial unpaired mindventral processes. This reduction of prolegs may reduce the friction of the abdomen with the substrate, and facilitate the locomotion of the larvae in the soil.

During their evolution from the Mesozoic (Byers and Thornhill 1983; Grimaldi and Engel 2005), the Mecoptera have evolved diverse larvae to adapt to various living habits. In most primitive Nannochoristidae the larvae stay in the substrate of streams and prey on the larvae of Chironomidae (Fraulob et al. 2012). In Boreidae the larvae of *Boreus* creep over plants and feed on fresh leaves (Cooper 1974), whereas the larvae of *Caurinus* feed in stem-mines or galleries of leafy liverworts (Russell 1982) or perhaps on other materials in recently deforested clear cuts (Sikes and Stockbridge 2013). In Bittacidae and Choristidae the larvae live on the surface of soil and feed on dead arthropods (Byers 1991; Tan and Hua 2008a). In Panorpidae the larvae live mostly in the soil, burrowing and concealing themselves while feeding on dead arthropods (Mampe and Neunzig 1965). In Panorpodidae, however, the larvae of *Panorpodes* are peculiar for their sedentary living habits and potentially live a root-feeding lifestyle. This is similar to the soil-dwelling and root-feeding larvae in Scarabaeidae, which are mostly eyeless and lack abdominal prolegs (Lawrence 1991). The consistency may indicate that the eyeless and proleg-reduced larval morphology are secondary adaptive traits to the soil-dwelling lifestyle.

In our rearing trial, the first instar larvae of *Panorpodes* fed on neither dead arthropods nor fresh leaves, although a darkened line in the alimentary canal was observed through the translucent trunk (L Jiang, unpublished data). The larvae we reared died eventually without molting, resulting in a failure to obtain the following instar larvae and pupae. This situation is similar to the observation of the confamilial *Brachypanorpa* (Byers 1997). The larval morphology and biology of later instars remain unknown.
